# Radiation-induced oral side effects in head and neck cancer: a scoping review and interdisciplinary recommendations

**DOI:** 10.1186/s12903-026-08100-4

**Published:** 2026-03-21

**Authors:** Till Gerlach, Giulia Brunello, Carina Tenbrink, Justus Schumacher, Jan Haussmann, Lisa Irschfeld, Judith Neuwahl, Livia Schmidt, Jörg Schipper, Christian Plettenberg, Lara Schorn, Norbert Kübler, Linea Weitz, Alexandra Ljimani, Gerald Antoch, Zahra Khosravi, Sara Röhrig, Holger Gottschlag, Petra Gierthmühlen, Frank Spitznagel, Martin Neukirchen, Andreas Künzel, Juliane Hörner-Rieber, Caroline Busch, Danny Jazmati

**Affiliations:** 1 Department of Oral Surgery, University Hospital of Düsseldorf, Medical Faculty, Heinrich Heine University Düsseldorf, Düsseldorf, Germany; 2 Department of Radiation Oncology, University Hospital Düsseldorf, Medical Faculty, Heinrich Heine University Düsseldorf, Düsseldorf, Germany; 3 Department of Neurosciences, Dentistry Section, University of Padova, Padova, 35128 Italy; 4 Department of Orthodontics and Dentofacial Orthopedics, Corporate Member of Freie Universität Berlin and Humboldt-Universität zu Berlin, Charité-Universitätsmedizin Berlin, Berlin, 14197 Germany; 5 Department of Otorhinolaryngology, Head and Neck Surgery, University Hospital Düsseldorf, Medical Faculty, Heinrich Heine University Düsseldorf, Düsseldorf, Germany; 6 Department of Oral and Maxillofacial Surgery, University Hospital Düsseldorf, Medical Faculty, Heinrich Heine University Düsseldorf, Düsseldorf, Germany; 7 Institute of Diagnostic and Interventional Radiology, University Hospital Düsseldorf, Medical Faculty, Heinrich Heine University Düsseldorf, Düsseldorf, Germany; 8 Department of Prosthodontics (Polyclinic for Dental Prosthetics), University Hospital Düsseldorf, Medical Faculty, Heinrich Heine University Düsseldorf, Düsseldorf, Germany; 9 Interdisciplinary Department of Palliative Care, University Hospital Düsseldorf, Medical Faculty, Heinrich Heine University Düsseldorf, Düsseldorf, Germany; 10 Department of Oral Medicine, Infection, and Immunity, Harvard School of Dental Medicine, Boston, USA

**Keywords:** Radiotherapy, Dentistry, Side effects, Osteoradionecrosis, Care model, Prevention, Head and neck cancer

## Abstract

**Introduction and objectives:**

Head and neck cancer remains a major global health challenge, with radiotherapy representing a central component of curative and palliative treatment. Despite advances in precision techniques, high-dose irradiation unavoidably affects adjacent oral structures, leading to a broad spectrum of side effects. These include dental caries, periodontitis, xerostomia, mucositis, candidiasis, trismus, dysphagia, and osteoradionecrosis—each with potential for chronic morbidity and diminished quality of life. This scoping review aims to summarize current evidence on oral side effects associated with head and neck radiotherapy, raise interdisciplinary awareness and propose a dental care model ensuring individualized therapies while focusing on prevention.

**Data:**

Radiation-induced oral effects are common, multifactorial, and often long-lasting. Caries and periodontitis are largely driven by hyposalivation and mucosal compromise, while trismus reflects dose-dependent impairment of the masticatory apparatus. Osteoradionecrosis, though less frequent, carries severe clinical consequences.

**Sources:**

A systematic literature search was conducted using PubMed for articles published between 1999 and November 2025. The search strategy combined the terms “head and neck cancer”, “radiotherapy” with the side effects mentioned above.

**Study selection:**

Studies were considered eligible if they examined oral side effects resulting from radiotherapy in patients with head and neck cancer, in total, 151 studies were included into the review.

**Results:**

We describe the epidemiology, underlying mechanisms, and clinical manifestations of common sequelae and provide a structured overview of preventive and therapeutic strategies.

**Clinical significance:**

The understanding, early identification and management of radiation-induced oral complications are essential to preserve function, maintain quality of life, and improve long-term outcomes for head and neck cancer patients.

## Introduction

Head and neck cancer ranks among the most common malignancies worldwide and is frequently treated with radiotherapy, either alone or in combination with other local or systemic therapies, or in the adjuvant setting following surgery [1]. While oncologic outcomes have improved substantially over recent decades, these advances are often tempered by the burden of treatment-related side effects [2]. In particular, the delivery of high-dose radiotherapy, typically in the range of 60–70 Gy – invariably exposes adjacent non-target tissues, including the dentition, salivary glands, oral mucosa, and masticatory apparatus [3–6].

The resulting complications are diverse in presentation, often multifactorial in origin, and carry a substantial risk of long-term persistence and often high symptom burden. Mucositis, xerostomia, radiation-induced caries, periodontitis, trismus, and osteoradionecrosis are among the most prevalent complications, collectively contributing to pain, functional impairment, nutritional deficits, and diminished quality of life. Many of these sequelae remain insufficiently anticipated in clinical pathways and are inconsistently addressed in follow-up care [4–7].

Modern radiotherapy techniques such as proton beam therapy have the potential to reduce long term treatment-related complications because of their more precise dose delivery and improved sparing of normal tissues [8]. The sharp distal dose fall-off of proton beam therapy may contribute to reduced radiation exposure of oral structures within the field of radiation and may support preservation of dental status [9–11]. However, prospective clinical studies are desirable to better understand how these dosimetric advantages translate into clinically meaningful outcomes [10]. Due to the scarcity of evidence this review will focus on radiotherapy based on photonic radiation.

This scoping review integrates current evidence on radiation-induced oral side effects in patients undergoing radiotherapy for head and neck cancer. We explore their underlying pathophysiological mechanisms, patterns of onset, and reported prevalence. Preventive and therapeutic strategies were reviewed considering evolving radiotherapy techniques and emerging supportive interventions. In addition, we highlight the clinical value of interdisciplinary models that systematically integrate dental care throughout the oncologic treatment trajectory. Finally, we outline future directions for translational research and clinical implementation, including biomarker-informed risk stratification and structured prospective data collection, with the aim of advancing toxicity mitigation and long-term survivorship outcomes.

## Methods

This scoping review was conducted in accordance with the PRISMA-ScR (Preferred Reporting Items for Systematic Reviews and Meta-Analyses extension for Scoping Reviews) guidelines [12] in Annals of Internal Medicine, and it followed methodological guidance from the Centre for Reviews and Dissemination (CRD) for evidence synthesis in health care. A formal review protocol was developed a priori to guide the review process.

The following research questions were formulated and to be answered by literature to be included in this review:

- What are the pathophysiologies, incidences, diagnostics, prevention measures, treatment options of specific side effects of radiotherapy?
- Can we define radiation dose limitations to a given side effect?
- Do the side effects influence each other?
- Are there risk factors regarding the onset of a given side effect?

### Search strategy

A systematic search of the electronic Database MEDLINE via PubMed and the Cochrane Library Database via the Cochrane Library website was conducted to identify relevant English-language studies published from 1999 up to November 2025. The search strategy included the search strings: radiotherapy caries, radiotherapy periodontitis, radiotherapy xerostomia, radiotherapy mucositis, radiotherapy tooth loss, radiotherapy candidiasis, Radiotherapy trismus, radiotherapy dysphagia, radiotherapy osteoradionecrosis,

Boolean operators and truncation were applied to optimize sensitivity and specificity across platforms. Study design filters for literature-, scoping- and systematic- reviews, meta-analyses, clinical trials and randomized controlled trials, were applied. A scoping review methodology was chosen to systematically map the breadth and heterogeneity of existing evidence in a field that spans multiple endpoints, clinical definitions, and methodological approaches.

### Eligibility criteria

Studies were eligible for inclusion if they:

- focused on oral toxicities associated with radiotherapy in head and neck cancer,
- reported original clinical data or provided a systematic review,
- were published in peer-reviewed journals,
- were written in English and available in full text.

The following were excluded:

- studies unrelated to radiotherapy in head and neck malignancies,
- articles without a clear focus on oral toxicities,
- editorials, letters, commentaries, conference abstracts without full text, or non-peer-reviewed literature.
- literature was published before 1999.

### Study selection

All retrieved records were imported into Zotero (v5.0) for de-duplication. Two reviewers (T. G., D. J.) independently screened titles and abstracts. Full-text articles of potentially eligible studies were retrieved and assessed against the inclusion criteria. Discrepancies were resolved through discussion or involvement of a third reviewer (C.B.). Both primary studies and secondary analyses (e.g., systematic reviews and meta-analyses) were included in this scoping review to comprehensively map the available literature. As this review aimed to provide an overview rather than perform quantitative synthesis, no pooled effect estimates were calculated.

To minimize duplication bias, systematic reviews and meta-analyses were used primarily to contextualize and summarize existing evidence rather than to duplicate extraction of primary outcome data. Where overlapping data were identified (e.g., prevalence estimates), priority was given to the more comprehensive source with the larger study population or most recent outcome. Primary studies were retained to capture novel findings, emerging evidence, or data not covered in secondary analyses.

### Data extraction and synthesis

A standardized data extraction template was developed to capture study characteristics, patient population, radiotherapy modalities, type and severity of oral complications, diagnostic methods, and therapeutic or preventive strategies. Data were categorized thematically according to the specific oral side effect, pathophysiological mechanism, and any reported clinical implications. Given the heterogeneity of study designs and outcomes, no formal meta-analysis was conducted. Instead, results were synthesized narratively and summarized in tables. A table of studies included in this review is provided in Table 1.

**Table 1:** Studies included in the review sorted alphabetically

Author	Year	Title	Journal	Article Type	Population size	Topic
Alhilali, et al.	2014	Osteoradionecrosis after radiation therapy for head and neck cancer: differentiation from recurrent disease with CT and PET/CT imaging	AJNR. American journal of neuroradiology	Review	63	Oseoradionecrosis
Ammajan, et al.	2013	Assessment of periodontal changes in patients undergoing radiotherapy for head and neck malignancy: A hospital-based study	Journal of Cancer Research and Therapeutics	Clinical Trial	29	Periodontitis
Balermpas, et al.	2022	Dental extraction, intensity-modulated radiotherapy of head and neck cancer, and osteoradionecrosis: A systematic review and meta-analysis	Strahlentherapie und Onkologie	Meta-analysis	875	Tooth loss
Barati, et al.	2025	Effectiveness of Photobiomodulation (low-level laser therapy) on treatment of oral mucositis (OM) induced by chemoradiotherapy in head and neck cancer patients.	Journal of photochemistry and photobiology. B, Biology	Clinical Trial	36	Mucositis
Bascil, et al.	2025	CARWL score as a predictor of radiation-induced periodontitis in locally advanced head and neck cancer undergoing concurrent chemoradiotherapy	Biomolecules & Biomedicine	Retrospective Study	67	Periodontitis
Batstone, et al.	2012	Platelet rich plasma for the prevention of osteoradionecrosis. A double blinded randomized cross over controlled trial	International Journal of Oral and Maxillofacial Surgery	Randomized Controlled Trial	22	Oseoradionecrosis
Bensadoun, et al.	2011	Oropharyngeal candidiasis in head and neck cancer patients treated with radiation: update 2011	Supportive Care in Cancer	Review	n.a.	Candidiasis
Blitzer, et al.	2025	Radiation-Therapy Related Salivary Dysfunction.	Seminars in radiation oncology	Review	n.a.	Xerostomia
Block, et al.	2022	Oral health and quality of life: findings from the Survey of Health, Ageing and Retirement in Europe	BMC oral health	Retrospective Study	59,048	Tooth loss
Brennan, et al.	2022	Tooth Failure Post-Radiotherapy in Head and Neck Cancer: Primary Report of the Clinical Registry of Dental Outcomes in Head and Neck Cancer Patients (OraRad) Study	International Journal of Radiation Oncology, Biology, Physics	Prospective Study	572	Tooth loss
Bueno, et al.	2013	Periodontal care in patients undergoing radiotherapy for head and neck cancer	Supportive Care in Cancer	Clinical Trial	25	Periodontitis
Chambers, et al.	2006	Clinical evaluation of the intraoral fluoride releasing system in radiation-induced xerostomic subjects. Part 2: Phase I study	Oral Oncology	Clinical Trial	22	Xerostomia
Charters, et al.	2025	Management of trismus after radiation therapy.	Current opinion in otolaryngology & head and neck surgery	Review	n.a.	Trismus
Chen, et al.	2023	Immune microenvironment: novel perspectives on bone regeneration disorder in osteoradionecrosis of the jaws	Cell and Tissue Research	Review	n.a.	Oseoradionecrosis
Chopra, et al.	2025	Strategies for obviation and management of trismus in oral cancer.	Current opinion in otolaryngology & head and neck surgery	Review	n.a.	Trismus
Chronopoulos, et al.	2018	Osteoradionecrosis of the jaws: definition, epidemiology, staging and clinical and radiological findings. A concise review	International Dental Journal	Review	n.a.	Oseoradionecrosis
Clarkson, et al.	2010	Interventions for treating oral mucositis for patients with cancer receiving treatment.	The Cochrane database of systematic reviews	Review	1505	Mucositis
Colella, et al.	2023	Interventions for the Prevention of Oral Mucositis in Patients Receiving Cancer Treatment: Evidence from Randomised Controlled Trials	Current Oncology	Review	n.a.	Mucositis
Conte, et al.	2025	Systematic Reviews on the Management of Xerostomia and Hyposalivation-An Umbrella Review.	Gerodontology	Systematic Review	n.a.	Xerostomia
da Silva et al.	2021	Photobiomodulation for mucosal repair in patients submitted to dental extraction after head and neck radiation therapy: a double-blind randomized pilot study.	Supportive care in cancer : official journal of the Multinational Association of Supportive Care in Cancer	Randomized Controlled Trial	40	Mucositis
da Silva Figueiredo et al.	2025	Materials and effects of intraoral device technologies for complication protection in head and neck cancer radiotherapy: a scoping review	BMC oral health	Scoping Review	n.a.	Tooth loss
Dai, et al.	2015	Surgical management of osteoradionecrosis of the jaws	The Journal of Craniofacial Surgery	Retrospective Study	120	Oseoradionecrosis
De Almaida Silva et al.	2024	The incidence of osteoradionecrosis of the jaws in oral cavity cancer patients treated with intensity-modulated radiotherapy: a systematic review and meta-analysis	Oral Surgery, Oral Medicine, Oral Pathology and Oral Radiology	Meta-analysis	3178	Oseoradionecrosis
De Carvalho et al.	2023	Oral care and the use of fluoride in the prevention of radiation-related caries: A scoping review	Oral Surgery, Oral Medicine, Oral Pathology and Oral Radiology	Scoping Review	n.a.	Caries
De Felice, et al.	2020	Xerostomia and Clinical Outcomes in Definitive Intensity Modulated Radiotherapy (IMRT) Versus Three-dimensional Conformal Radiotherapy (3D-CRT) for Head and Neck Squamous Cell Carcinoma: A Meta-analysis	in vivo	Meta-analysis	213	Xerostomia
Delanian, et al.	2005	Major healing of refractory mandible osteoradionecrosis after treatment combining pentoxifylline and tocopherol: a phase II trial.	Head & neck	Clinical Trial	18	Oseoradionecrosis
Deshpande, et al.	2015	Osteoradionecrosis of the mandible: through a radiologist’s eyes.	Clinical radiology	Review	n.a.	Oseoradionecrosis
El Harram et al.	2025	Radiation-Induced Caries: Exploring the Pathway to Manage the Challenge.	Cureus	Review	n.a.	Caries
El Hawari et al.	2022	Protective and positioning devices in maxillofacial prosthodontics and radiotherapy: Overview	Technical Innovations & Patient Support in Radiation Oncology	Review	n.a.	Oseoradionecrosis
El-Rabbany, et al.	2019	Interventions for preventing osteoradionecrosis of the jaws in adults receiving head and neck radiotherapy	Cochrane Database of Systematic Reviews	Systematic Review	342	Oseoradionecrosis
Epstein, et al.	2012	Oral complications of cancer and cancer therapy: From cancer treatment to survivorship	CA: A Cancer Journal for Clinicians	Review	n.a.	Periodontitis
Epstein, et al.	1999	A double-blind crossover trial of Oral Balance gel and Biotene^®^ toothpaste versus placebo in patients with xerostomia following radiation therapy	Oral Oncology	Randomized Controlled Trial	19	Xerostomia
Fan, et al.	2025	Comprehensive Update on Implants in Patients With Head and Neck Cancer (2021–2024): Systematic Review and Meta-Analysis of the Impact of Radiotherapy and Chemotherapy on Implant Survival	Clinical Oral Implants Research	Meta-analysis	836	Tooth loss
Forner, et al.	2022	Hyperbaric oxygen treatment of mandibular osteoradionecrosis: Combined data from the two randomized clinical trials DAHANCA-21 and NWHHT2009-1	Radiotherapy and Oncology: Journal of the European Society for Therapeutic Radiology and Oncology	Randomized Controlled Trial	65	Oseoradionecrosis
Fregnani, et al.	2016	IMRT delivers lower radiation doses to dental structures than 3DRT in head and neck cancer patients	Radiation oncology (London, England)	Retrospective Study	80	Radiotherapy
Funahara, et al.	2022	Effects of a miconazole oral patch on preventing development of oral candidiasis in patients with head and neck cancer undergoing radiotherapy: results of a preliminary study quantifying the prevalence of Candida albicans in saliva	Supportive Care in Cancer: Official Journal of the Multinational Association of Supportive Care in Cancer	Clinical Trial	28	Candidiasis
Furness, et al.	2011	Interventions for the management of dry mouth: topical therapies.	The Cochrane database of systematic reviews	Systematic Review	1597	Xerostomia
García-Chías, et al.	2019	Prevalence of oral side effects of chemotherapy and its relationship with periodontal risk: a cross sectional study	Supportive Care in Cancer: Official Journal of the Multinational Association of Supportive Care in Cancer	Cross-sectional Study	369	Periodontitis
Ghorbani, et al.	2021	Effect of Ozonated Water on Oral Mucositis and Pain Induced by Head and Neck Radiotherapy: a Cross-sectional Study	Archives of neuroscience	Cross-sectional Study	93	Mucositis
Goel, et al.	2010	Use of positioning stents in lingual carcinoma patients subjected to radiotherapy.	The International journal of prosthodontics	Clinical Trial	48	Positioning stents
Goh, et al.	2023	The dental management of patients irradiated for head and neck cancer	British Dental Journal	Review	n.a.	Caries
Gomes-Silva, et al.	2021	Impact of radiation on tooth loss in patients with head and neck cancer: a retrospective dosimetric-based study	Oral Surgery, Oral Medicine, Oral Pathology and Oral Radiology	Retrospective Study	66	Tooth loss
Gomes, et al.	2025	Dysphagia, nutritional status, and quality of life in patients with head and neck cancer undergoing radiotherapy alone or combined with chemotherapy: an observational study	BMC cancer	Observarional study	47	Dysphagia
Gorsky, et al.	2004	The efficacy of pilocarpine and bethanechol upon saliva production in cancer patients with hyposalivation following radiation therapy.	Oral surgery, oral medicine, oral pathology, oral radiology, and endodontics	Clinical Trial	42	Xerostomia
Gossweiler, et al.	2025	TOPICAL FLUORIDES MAY PREVENT RADIATION CARIES IN ADULTS UNDERGOING HEAD AND NECK RADIOTHERAPY	The Journal of Evidence-Based Dental Practice	Meta-analysis	n.a.	Caries
Govender, et al.	2024	Post-Radiotherapy Dysphagia in Head and Neck Cancer: Current Management by Speech-Language Pathologists.	Current treatment options in oncology	Review	n.a.	Dysphagia
Grepl, et al.	2020	The Changes in Pharyngeal Constrictor Muscles Related to Head and Neck Radiotherapy: A Systematic Review	Technology in Cancer Research & Treatment	Systematic Review	228	Dysphagia
Gupta, et al.	2015	Radiation-induced dental caries, prevention and treatment - A systematic review	National Journal of Maxillofacial Surgery	Systematic Review	n.a.	Caries
Hague, et al.	2018	Prospective evaluation of relationships between radiotherapy dose to masticatory apparatus and trismus	Acta Oncologica	Prospective Study	20	Trismus
Held, et al.	2021	3D-printed individualized tooth-borne tissue retraction devices compared to conventional dental splints for head and neck cancer radiotherapy: a randomized controlled trial	Radiation oncology (London, England)	Randomized Controlled Trial	34	Positioning stents
Huang, et al.	2022	Impact on xerostomia for patients with nasopharyngeal carcinoma treated with superficial parotid lobe-sparing intensity-modulated radiation therapy (SPLS-IMRT): A prospective phase II randomized controlled study.	Journal of Clinical Oncology	Randomized Study	90	Xerostomia
Huynh, et al.	2024	Radiation-induced long-term dysphagia in survivors of head and neck cancer and association with dose-volume parameters	Radiotherapy and Oncology: Journal of the European Society for Therapeutic Radiology and Oncology	Cross-sectional Study	239	Dysphagia
Jakobsen, et al.	2024	Mesenchymal Stem/Stromal Cell Therapy for Radiation-Induced Xerostomia in Previous Head and Neck Cancer Patients: A Phase II Randomized, Placebo-Controlled Trial	Clinical Cancer Research: An Official Journal of the American Association for Cancer Research	Randomized Controlled Trial	120	Xerostomia
Jawad, et al.	2015	A review of dental treatment of head and neck cancer patients, before, during and after radiotherapy: part 1	British Dental Journal	Review	n.a.	Radiotherpy side effects
Jawad, et al.	2015	A review of dental treatment of head and neck cancer patients, before, during and after radiotherapy: part 2	British Dental Journal	Review	n.a.	Radiotherpy side effects
Jiang, et al.	2024	The effects of an integrated supportive programme on oral health in patients with head and neck cancer undergoing radiotherapy: A randomized controlled trial	International Journal of Dental Hygiene	Randomized Controlled Trial	92	Caries
Kang, et al.	2022	Progression and postoperative complications of osteoradionecrosis of the jaw: a 20-year retrospective study of 124 non-nasopharyngeal cancer cases and meta-analysis	BMC Oral Health	Meta-analysis	124	Oseoradionecrosis
Kaur, et al.	2023	Safety and efficacy of oral pilocarpine in radiation-induced xerostomia in oropharyngeal carcinoma patients	Journal of Cancer Research and Therapeutics	Clinical Trial	60	Xerostomia
Khaw, et al.	2014	Radiation-induced oral mucositis and periodontitis – proposal for an inter‐relationship	Oral Diseases	Review	n.a.	Periodontitis
Kielbassa, et al.	2006	Radiation-related damage to dentition	The Lancet Oncology	Review	n.a.	Caries
Kim, et al.	2017	Effects of an oral health promotion program in head and neck cancer patients receiving radiation therapy: results of a prospective cohort study	International journal of radiation oncology biology physics	Prospective Study	84	Caries
Kolokythas, et al.	2019	Management of osteoradionecrosis of the jaws with pentoxifylline-tocopherol: a systematic review of the literature and meta-analysis	International Journal of Oral and Maxillofacial Surgery	Systematic Review and Meta-analysis	211	Oseoradionecrosis
Kovarik, et al.	2024	Timing of development of osteoradionecrosis post head and neck radiotherapy: does a safe time interval exist for dental extraction?	Strahlentherapie und Onkologie	Retrospective Study	1608	Oseoradionecrosis
Kraaijenga, et al.	2015	Evaluation of long term (10-years+) dysphagia and trismus in patients treated with concurrent chemo-radiotherapy for advanced head and neck cancer	Oral Oncology	Clinical Trial	22	Dysphagia
Kün-Darbois, et al.	2021	Medication-related osteonecrosis and osteoradionecrosis of the jaws: Update and current management	Morphologie	Review	n.a.	Oseoradionecrosis
Lakshman, et al.	2015	Evaluation of effect of transcutaneous electrical nerve stimulation on salivary flow rate in radiation induced xerostomia patients: a pilot study	Journal of cancer research and therapeutics	Prospective Study	40	Xerostomia
Lalla, et al.	2010	A systematic review of oral fungal infections in patients receiving cancer therapy	Supportive Care in Cancer: Official Journal of the Multinational Association of Supportive Care in Cancer	Systematic Review	n.a.	Candidiasis
Lee, et al.	2022	Pathogenesis and Amelioration of Radiation-Induced Oral Mucositis	Current Treatment Options in Oncology	Review	n.a.	Mucositis
Lee, et al.	2021	The effect of comprehensive oral care program on oral health and quality of life in patients undergoing radiotherapy for head and neck cancer: A quasi-experimental case-control study	Medicine	Clinical Trial	61	
Lee, et al.	2018	Randomised feasibility study to compare the use of Therabite ^®^ with wooden spatulas to relieve and prevent trismus in patients with cancer of the head and neck	British Journal of Oral and Maxillofacial Surgery	Randomized Study	71	Trismus
Lieshout, et al.	2014	The effect of radiotherapy on dental hard tissue—a systematic review	Clinical Oral Investigations	Systematic Review	n.a.	Caries
Lin, et al.	2023	Hyperbaric oxygen therapy for late radiation tissue injury	The Cochrane Database of Systematic Reviews	Systematic Review	1071	Oseoradionecrosis
Lopez-Garzon, et al.	2024	Efficacy of photobiomodulation therapy combined with mobile health education in patients with head and neck cancer suffering from chronic xerostomia after radiotherapy: protocol for a three-arm, randomised, placebo-controlled, double-blinded study	BMJ Open	Randomized Study	20	Xerostomia
Lu et al.	2019	Direct radiation-induced effects on dental hard tissue	Radiation Oncology	in vitro study	60	Caries
Luka, et al.	2024	PREVENTING CARIES AFTER RADIOTHERAPY TO THE HEAD AND NECK REGION – A SYSTEMATIC REVIEW	Journal of Evidence-Based Dental Practice	Systematic Review	355	Caries
Madan, et al.	2008	The effect of three mouthwashes on radiation-induced oral mucositis in patients with head and neck malignancies: a randomized control trial.	Journal of cancer research and therapeutics	Randomized Study	76	Mucositis
Majid, et al.	2024	Are prophylactic antibiotics effective in preventing osteoradionecrosis after high-risk dental extractions?	Evidence-Based Dentistry	Review	1520	Oseoradionecrosis
Malouf, et al.	2003	Influence of parotid-sparing radiotherapy on xerostomia in head and neck cancer patients	Cancer Detection and Prevention	Clinical Trial	93	Xerostomia
Marchand, et al.	2020	Pilocarpine for Radiotherapy-Induced Dry Mouth and Dry Eyes: A Review of Clinical Effectiveness, Cost-Effectiveness, and Guidelines	CADTH Rapid Response Reports	Review		Xerostomia
Maret, et al.	2024	Consequences of hyposalivation in relation to cancer treatment and early management of radiation-induced caries: case reports	British Dental Journal	Case Report	n.a.	Xerostomia
Maria, et al.	2017	Radiation-Induced Oral Mucositis	Frontiers in Oncology	Review	n.a.	Mucositis
Marques, et al.	2004	Periodontal Changes in Patients Undergoing Radiotherapy	Journal of Periodontology	Clinical Trial	27	Periodontitis
Martins, et al.	2021	Photobiomodulation reduces the impact of radiotherapy on oral health-related quality of life due to mucositis-related symptoms in head and neck cancer patients	Lasers in medical science	Clinical Trial	48	Mucositis
Mastroianni, et al.	2025	Concordance of the World Health Organization, Oral Assessment Guide, and Tardieu Scales for Assessment of Oral Mucositis and Oral Disorders in Palliative Care Patients	Journal of Palliative Medicine	Prospective Study	77	Palliative Care
McComb, et al.	2002	A clinical comparison of glass ionomer, resin-modified glass ionomer and resin composite restorations in the treatment of cervical caries in xerostomic head and neck radiation patients.	Operative dentistry	Observarional study	45	Caries
Mercadante, et al.	2025	A systematic review of salivary gland hypofunction and/or xerostomia induced by non-surgical cancer therapies: prevention strategies.	Supportive care in cancer : official journal of the Multinational Association of Supportive Care in Cancer	Systematic Review	n.a.	Xerostomia
Mercadante, et al.	2017	Interventions for the management of radiotherapy-induced xerostomia and hyposalivation: A systematic review and meta-analysis	Oral Oncology	Meta-analysis	1732	Xerostomia
Miyamoto, et al.	2021	Clinical Diagnostic Imaging Study of Osteoradionecrosis of the Jaw: A Retrospective Study	Journal of Clinical Medicine	Retrospective Study	54	Oseoradionecrosis
Mohandas, et al.	2025	Comparative evaluation of the efficacy of herbal and benzydamine mouthwashes in preventing radiation-induced oral mucositis among head and neck cancer patients: a systematic review and network meta-analysis.	Evidence-based dentistry	Meta-analysis	n.a.	Mucositis
Moon, et al.	2017	Incidence of, and risk factors for, mandibular osteoradionecrosis in patients with oral cavity and oropharynx cancers	Oral Oncology	Retrospective Study	252	Oseoradionecrosis
Moore, et al.	2025	A prospective clinical study of the influence of dental and salivary gland radiation dose on dental caries development in patients with head and neck cancer	Journal of Dentistry	Prospective Study	151	Xerostomia
Moreno, et al.	2025	International Expert-Based Consensus Definition, Classification Criteria, and Minimum Data Elements for Osteoradionecrosis of the Jaw: An Interdisciplinary Modified Delphi Study.	International journal of radiation oncology, biology, physics	Delphi Study	n.a.	Oseoradionecrosis
Motallebnejad, et al.	2008	The effect of topical application of pure honey on radiation-induced mucositis: a randomized clinical trial	Journal of contemporary dental practice	Randomized Study	40	Mucositis
Mukesh, et al.	2013	Role of fluconazole in the prevention of radiation induced mucositis in head and neck cancer patients	European journal of cancer	Randomized Controlled Trial	155	Mucositis
Murphy et al.	2025	Xerostomia: a silent burden for people receiving palliative care - a qualitative descriptive study	BMC palliative care	Review	n.a.	Xerostomia
Nevens, et al.	2017	Can sparing of the superficial contralateral parotid lobe reduce xerostomia following radiotherapy for head and neck cancer?	The British Journal of Radiology	Prospective Study	88	Xerostomia
Nuchit, et al.	2020	Alleviation of dry mouth by saliva substitutes improved swallowing ability and clinical nutritional status of post-radiotherapy head and neck cancer patients: a randomized controlled trial	Supportive Care in Cancer	Randomized Controlled Trial	62	Xerostomia
Nutting, et al.	2023	Dysphagia-optimised intensity-modulated radiotherapy versus standard intensity-modulated radiotherapy in patients with head and neck cancer (DARS): a phase 3, multicentre, randomised, controlled trial	The Lancet. Oncology	Randomized Study	118	Dysphagia
Obermeier, et al.	2025	Antiresorptive therapy in combination with radiation results in enhanced risk for necrosis and associated complicatifions	Oral Surgery, Oral Medicine, Oral Pathology and Oral Radiology	Observarional study	17	MRONJ
Paetkau, et al.	2024	Pharyngeal Constrictor Dose-Volume Histogram Metrics and Patient-Reported Dysphagia in Head and Neck Radiotherapy	Clinical Oncology (Royal College of Radiologists (Great Britain))	Retrospective Study	88	Dysphagia
Pak, et al.	2025	Osteoradionecrosis of the Jaw-Thinking Outside the Box, a Review of Emerging Treatment Paradigms.	The Journal of craniofacial surgery	Review	n.a.	Oseoradionecrosis
Palma, et al.	2020	Leukocyte- and platelet-rich fibrin does not provide any additional benefit for tooth extraction in head and neck cancer patients post-radiotherapy: a randomized clinical trial.	Medicina oral, patologia oral y cirugia bucal	Randomized Controlled Trial	23	Tooth Extration
Papas, et al.	2008	Caries clinical trial of a remineralising toothpaste in radiation patients	Gerodontology	Clinical Trial	44	Caries
Pathak, et al.	2024	A Prospective Study of the Incidence of Chronic Xerostomia and the Quality of Life in Patients Undergoing Radiotherapy for Head and Neck Malignancies with IMRT or VMAT Techniques	The Gulf Journal of Oncology	Prospective Study	80	Xerostomia
Pauli, et al.	2015	Treating trismus: a prospective study on effect and compliance to jaw exercise therapy in head and neck cancer	Head & neck	Prospective Study	50	Trismus
Peterson, et al.	2024	Prevention and Management of Osteoradionecrosis in Patients With Head and Neck Cancer Treated With Radiation Therapy: ISOO-MASCC-ASCO Guideline.	Journal of clinical oncology : official journal of the American Society of Clinical Oncology	Guideline	n.a.	Oseoradionecrosis
Petersson, et al.	2025	Preventing radiation-induced dysphagia and trismus in head and neck cancer-A randomized controlled trial	Head & Neck	Randomized Controlled Trial	89	Dysphagia
Pettersson, et al.	2024	Decreased Rates of Radiation-induced Trismus and Lowered Mastication Structure Doses in Patients Treated for Head and Neck Cancer During the Last Two Decades	Clinical Oncology	Retrospective Study	121	Trismus
Pinna, et al.	2015	Xerostomia induced by radiotherapy: an overview of the physiopathology, clinical evidence, and&nbsp;management of the oral damage	Therapeutics and Clinical Risk Management	Review	n.a.	Xerostomia
Pitchaimuthu, et al.	2025	78115929-1819 - Trismus Prevention in Head and Neck Cancer Patient Undergoing Radiotherapy	International Journal of Oral and Maxillofacial Surgery	Randomized Controlled Trial	30	Trismus
Pontes, et al.	2022	DENTAL TREATMENT ASSOCIATED WITH PHOTO MODULATION THERAPY IN MUCOSITIS AND INFLAMMATORY BIOMARKERS IN PATIENTS WITH HEAD AND NECK CANCER.	Supportive Care in Cancer	Randomized Controlled Trial	103	Mucositis
Pow, et al.	2006	Xerostomia and quality of life after intensity-modulated radiotherapy vs. conventional radiotherapy for early-stage nasopharyngeal carcinoma: initial report on a randomized controlled clinical trial.	International journal of radiation oncology, biology, physics	Randomized Controlled Trial	51	Xerostomia
Quah, et al.	2024	Efficacy of adjunctive modalities during tooth extraction for the prevention of osteoradionecrosis: A systematic review and meta-analysis	Oral Diseases	Meta-analysis	1520	Tooth Extration
Rastogi, et al.	2017	Role of benzydamine hydrochloride in the prevention of oral mucositis in head and neck cancer patients treated with radiotherapy (> 50 Gy) with or without chemotherapy	Supportive Care in Cancer: Official Journal of the Multinational Association of Supportive Care in Cancer	Clinical Trial	120	Mucositis
Riley, et al.	2015	Interventions for preventing oral mucositis in patients with cancer receiving treatment: oral cryotherapy.	The Cochrane database of systematic reviews	Review	n.a.	Mucositis
Saghafi, et al.	2025	Risk and Health Factors for Temporomandibular Disorders Following Radiotherapy for Head and Neck Cancer.	Journal of oral rehabilitation	Randomized Controlled Trial	58	Trismus
Salimi, et al.	2021	Trans-cutaneous electrical nerve stimulation to treat dry mouth (xerostomia) following radiotherapy for head and neck cancer. A systematic review	Annals of Medicine & Surgery	Systematic Review	n.a.	Xerostomia
Satheeshkumar, et al.	2010	Effectiveness of triclosan in the management of radiation-induced oral mucositis: A randomized clinical trial	Journal of Cancer Research and Therapeutics	Randomized Study	24	Mucositis
Schanne, et al.	2024	Effect of dose to parotid ducts on Sticky Saliva and Xerostomia in radiotherapy of head and neck squamous cell carcinoma	Radiation Oncology	Retrospective Study	99	Xerostomia
See et al.	2018	Dental extractions for preradiation dental clearance and incidence of osteoradionecrosis in patients with nasopharyngeal carcinoma treated with intensity-modulated radiotherapy	Journal of Investigative and Clinical Dentistry	Retrospective Study	231	Tooth Extration
Serra, et al.	2024	Oral hygiene care and the management of oral symptoms in patients with cancer in palliative care: a mixed methods systematic review protocol	JBI evidence synthesis	Systematic Review	n.a.	Palliative Care
Shah, et al.	2020	Effectiveness of curcumin mouthwash on radiation-induced oral mucositis among head and neck cancer patients: A triple-blind, pilot randomised controlled trial.	Indian journal of dental research : official publication of Indian Society for Dental Research	Randomized Controlled Trial	74	Mucositis
Shao, et al.	2020	Exercise therapy for cancer treatment-induced trismus in patients with head and neck cancer: A systematic review and meta-analysis of randomized controlled trials.	Radiotherapy and oncology : journal of the European Society for Therapeutic Radiology and Oncology	Systematic Review and Meta-analysis	733	Trismus
Shodo, et al.	2025	Oropharyngeal candidiasis during radiotherapy for head and neck cancer: an observational study on prevalence, pain, and risk factors	European archives of oto-rhino-laryngology: official journal of the European Federation of Oto-Rhino-Laryngological Societies (EUFOS): affiliated with the German Society for Oto-Rhino-Laryngology - Head and Neck Surgery	Retrospective Study	65	Candidiasis
Shrivastava, et al.	2017	The Effect Of Chlorhexidine Mouthwash On Chemoradiotherapy Induced Mucositis In Patients Of Oral Cavity And Oropharyngeal Carcinoma	Journal of cancer research and therapeutics	Clinical Trial	60	Mucositis
Silva, et al.	2009	Patterns of Demineralization and Dentin Reactions in Radiation-Related Caries	Caries Research	Review	n.a.	Caries
Sim, et al.	2019	Anticariogenic efficacy of a saliva biomimetic in head-and-neck cancer patients undergoing radiotherapy.	Australian dental journal	Randomized Controlled Trial	24	Caries
Singh, et al.	2022	Osteoradionecrosis of the jaw: A mini review	Frontiers in Oral Health	Review	n.a.	Oseoradionecrosis
Sohn, et al.	2018	Effects of professional oral hygiene care in patients with head-and-neck cancer during radiotherapy: A randomized clinical trial	Indian Journal of Dental Research	Randomized Controlled Trial	40	Caries
Soutome, et al.	2020	Prevention of dental caries by regular overnight application of a low-concentration fluoride gel loaded in a custom tray in patients undergoing radiotherapy for head and neck cancer: a preliminary study	Indian journal of dental research	Prospective Study	13	Caries
Sroussi, et al.	2017	Common oral complications of head and neck cancer radiation therapy: mucositis, infections, saliva change, fibrosis, sensory dysfunctions, dental caries, periodontal disease, and osteoradionecrosis	Cancer Medicine	Review	n.a.	Radiotherpy side effects
Strojan, et al.	2017	Treatment of late sequelae after radiotherapy for head and neck cancer	Cancer Treatment Reviews	Review	n.a.	Radiotherpy side effects
Sueira, et al.	2025	Oral toxicities associated with immunotherapy and targeted therapy in cancer treatment	Oral Oncology Reports	Review	n.a.	Radiotherpy side effects
Tang, et al.	2011	A Randomized Prospective Study of Rehabilitation Therapy in the Treatment of Radiation-induced Dysphagia and Trismus	Strahlentherapie und Onkologie	Randomized Controlled Trial	43	Trismus and Dysphagia
Taweechaisupapong, et al.	2006	Efficacy of pilocarpine lozenge for post-radiation xerostomia in patients with head and neck cancer.	Australian dental journal	Randomized Controlled Trial	33	Xerostomia
Taylor, et al.	2025	Toxicities in long-term survivors of head and neck cancer—A multi-national cross-sectional analysis	International Journal of Cancer	Cross-sectional Study	1094	Radiotherpy side effects
Topkan, et al.	2023	Review of Osteoradionecrosis of the Jaw: Radiotherapy Modality, Technique, and Dose as Risk Factors	Journal of Clinical Medicine	Review	n.a.	Oseoradionecrosis
Tu, et al.	2024	The influence of different radiotherapy doses on the mechanical properties and microstructure of the enamel and dentin of human premolar teeth	Strahlentherapie und Onkologie	in vitro study	25	Caries
Uhlig, et al.	2024	Exploring the integration of dentistry within a multidisciplinary palliative care team: does bedside dental care improve quality of life and symptom burden in inpatient palliative care patients?	Supportive Care in Cancer: Official Journal of the Multinational Association of Supportive Care in Cancer	Prospective Study	103	Palliative Care
Urquhart, et al.	2022	Effect of preradiation dental intervention on incidence of osteoradionecrosis in patients with head and neck cancer	The Journal of the American Dental Association	Meta-analysis	113,812	Oseoradionecrosis
Van Dyke et al.	2005	Risk factors for periodontitis	Journal of the International Academy of Periodontology	Review	n.a.	Periodontitis
van Rijn-Dekker et al.	2024	Clinical Introduction of Stem Cell Sparing Radiotherapy to Reduce the Risk of Xerostomia in Patients with Head and Neck Cancer	Cancers	Clinical Trial	30	Xerostomia
Verro, et al.	2025	Beyond Conventional Treatments: The Role of Complementary Therapies in Head and Neck Cancer.	Cancers	Review	n.a.	Xerostomia
Vianna Camolesi et al.	2025	Photobiomodulation for the prevention of oral side effects secondary to head and neck cancer therapy: results of a randomised, single-blind clinical trial.	Oral oncology	Randomized Study	53	Radiotherpy side effects
Villa, et al.	2025	Addressing Pain in Oral Mucositis: Narrative Review of Current Practices and Emerging Treatments.	Journal of pain research	Review	n.a.	Mucositis
Watson, et al.	2024	Development and Standardization of an Osteoradionecrosis Classification System in Head and Neck Cancer: Implementation of a Risk-Based Model	Journal of Clinical Oncology: Official Journal of the American Society of Clinical Oncology	Retrospective Study	2732	Oseoradionecrosis
Watters, et al.	2019	Prevalence of trismus in patients with head and neck cancer: A systematic review with meta-analysis	Head & Neck	Meta-analysis	2786	Trismus
Worthington, et al.	2010	Interventions for treating oral candidiasis for patients with cancer receiving treatment.	The Cochrane database of systematic reviews	Systematic Review	940	Candidiasis
Yfanti, et al.	2023	Radiologic findings of osteonecrosis, osteoradionecrosis, osteomyelitis and jaw metastatic disease with cone beam CT	European Journal of Radiology	Retrospective Study	335	Radiology
Zhang, et al.	2024	Evidence summary on managing radiotherapy-induced oral mucositis in patients with head and neck cancer	Asia-Pacific Journal of Oncology Nursing	Review	n.a.	Mucositis

### Methodological appraisal

As the primary goal of a scoping review was to provide an overview of available evidence rather than assess intervention efficacy, no formal risk of bias assessment was performed. However, we prioritized studies with transparent methodology, appropriate cohort sizes and clear clinical relevance. A more detailed analysis of the limitations of the evidence base is given in the Limitations section.

## Results

### Dental caries

**Fig. 1:**
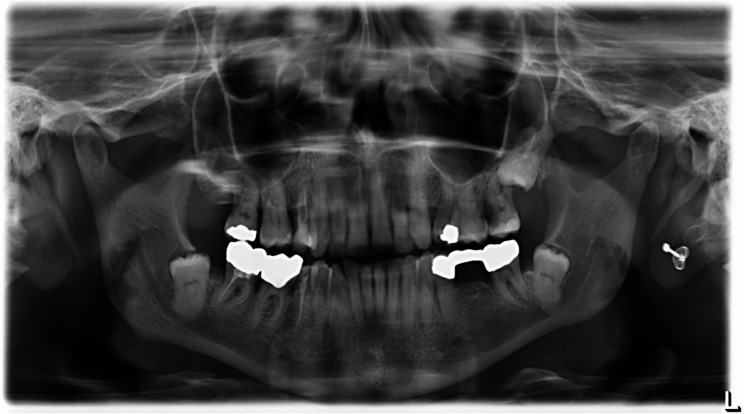
Panoramic radiograph of a patient diagnosed with squamous cell carcinoma localized in the floor of the mouth before recieving radiotherapy, multiple restaurations are visible on the lower molars and on teeth 17 and 25 in metallic opacity. We thank the Oral Surgery Department of Düsseldorf University Hospital for providing these radiographs

**Fig. 2:**
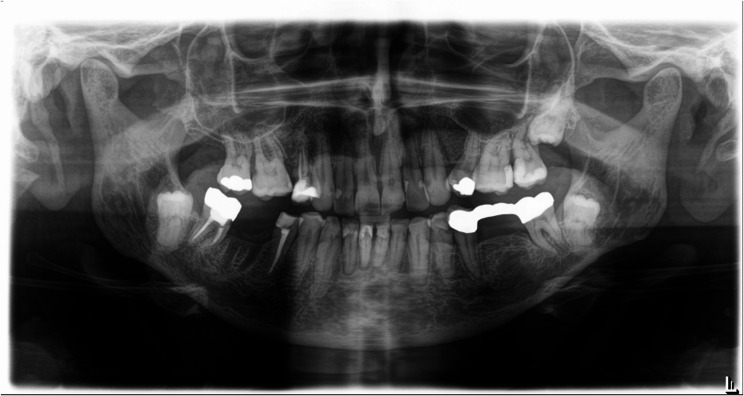
Panoramic radiograph of the same patient 5 years after radiotherapy with new restorations especially in the lower front and upper front teeth as well as a fresh alveolar wound after extraction of tooth 46

**Fig. 3:**
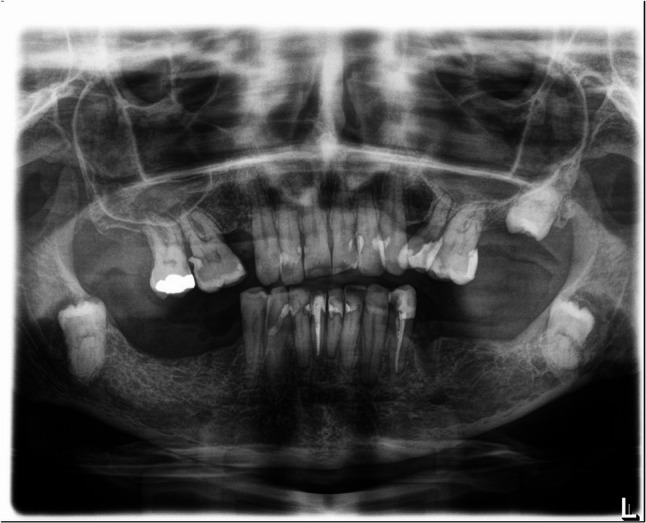
Panoramic radiograph of the same patient 7 years after radiotherapy, multiple new restorations in upper front and premolar regions, endodontic treatment of teeth 35 and 41 and extractions of teeth 14, 27, 37, 45 and 47 have been performed, significant bone loss is visible in the molar regions of the mandible compared to the earlier radiographs

Unlike caries in non-irradiated individuals, radiation-induced caries primarily affects smooth tooth surfaces, including labial and cervical regions [13, 14]. Although cohort-specific differences are reported, the overall prevalence of radiation caries exceeds 25%, largely associated with xerostomia following radiotherapy [15, 16]. Figures 1, 2 and 3 show exemplary panoramic radiographs illustrating the progression of dental status over time in a patient undergoing radiotherapy. Multiple new fillings appeared at the front teeth, as well as multiple molars and premolars were extracted over time (Figs. 1, 2 and 3).

Mucositis and hyposalivation, both recognized sequelae of radiotherapy, are significant contributors to caries risk. Moreover, oral hygiene practices frequently decline due to therapy-related discomfort, further compounding the risk of carious lesions [14, 16, 17].

Similar to conventional dental caries, radiation caries is a multifactorial condition driven by an imbalance between demineralization and remineralization. This disequilibrium is exacerbated by radiation-induced hyposalivation, mucosal inflammation, and compromised oral hygiene [14, 16, 18–20].

Emerging evidence indicates that radiation impairs the integrity of dental hard tissues, particularly at the dentin–enamel junction. While the clinical relevance of these microstructural changes to caries formation remains limited, they contribute to overall tooth fragility [21–23]. Enamel changes are observed from cumulative doses of 30–70 Gy, whereas dentinal alterations may occur from exposures as low as 10 Gy [24].

Additionally, patients may report heightened dentinal hypersensitivity – likely due to reduced salivary coverage and diminished buffering capacity [14].

Preventive strategies aim to restore the balance in favor of remineralization. Topical fluoride application remains the cornerstone of prevention, with additional benefit observed from saliva substitutes enriched with calcium ions [13, 25–33]. The efficacy of such interventions depends on consistent use; single applications have negligible clinical impact [25]. Integrative care models that promote patient engagement and adherence to preventive routines can reduce caries incidence [34, 35].

Pre-radiotherapy dental evaluation is relevant for identifying and managing existing oral pathologies and eliminating potential sources of infection. This approach helps minimize the risk of complications such as osteoradionecrosis and supports the implementation of preventive strategies against radiation-related caries [36, 37].

Management of radiation-induced caries is notably more challenging than that of conventional caries [16]. Restorative approaches commonly employ either resin composites or glass ionomer cements. A clinical trial found, while resin composites offer superior structural durability, glass ionomer restorations may confer greater secondary preventive benefit, likely due to sustained fluoride release [38].

In addition to established restorative approaches, the role of professional oral prophylaxis and patient education should not be underestimated [39]. Consistent reinforcement of self-care behaviors, supported by structured dental follow-up, has been shown to improve adherence to fluoride use and reduce caries incidence by a case control study [29].

### Periodontitis

Periodontitis is characterized by progressive loss of tooth-supporting structures, including both the alveolar bone and periodontal ligament. While the structural integrity of the tooth may remain intact, the weakening of its anchoring apparatus ultimately compromises stability and can result in tooth loss. Radiotherapy has been implicated as a contributing factor to this form of attachment loss, particularly within the irradiated field [40].

Prevalence for radiotherapy induced periodontitis is ranging from 50% [41] to 70% [42]. Notably, the mandible appears to be disproportionately affected compared to the maxilla, as evidenced by increased rates of attachment loss [42, 43]. This observation underscores the importance of jaw-specific considerations during radiotherapy planning. Quantification of attachment loss – typically calculated as the sum of gingival recession and probing depth – demonstrates that radiotherapy can induce a mean loss of at least 0.2 mm in 61% of mandibular and 34% of maxillary teeth [43]. On top of the clinical assessment of periodontitis, laboratory biomarkers such as the C-reactive protein-to-albumin ratio to weight loss (CARWL) could be a future direction towards predicting high risk of periodontitis onset on irradiated patients [44]. Collectively, these findings support the interpretation of periodontitis as a common and clinically significant side effect of radiotherapy, meriting proactive assessment and longitudinal monitoring by dental care providers [42, 43].

Given the irreversible nature of periodontal attachment loss, preventive strategies are paramount. Oral hygiene remains the foundation of prophylaxis, encompassing patient education on self-care techniques such as routine toothbrushing and the use of interdental cleaning devices [45], as well as regular professional dental cleanings [35]. Dose-reduction strategies to spare the periodontium may be considered during radiotherapy planning; however, these are often constrained by the anatomical positioning of the tumor. The multifactorial etiology of periodontitis is well recognized, with modifiable risk factors – including smoking, poorly controlled diabetes, and inadequate oral hygiene – playing pivotal roles in both onset and progression. Mitigation of these factors may aid in prevention or enhance treatment response [46].

In cases where periodontitis is already established, mechanical debridement – namely scaling and root planing – remains the primary therapeutic approach. The objective is the thorough removal of plaque and subgingival calculus. Importantly, this intervention has demonstrated efficacy even when administered during ongoing radiotherapy in a clinical trial [45].

### Xerostomia

Xerostomia, or mouth dryness, is a well-recognized sequela of radiotherapy to the head and neck region, with severity closely linked to cumulative dose exposure to the salivary glands. Among these, the parotid gland plays a central role due to its predominant contribution to resting salivary flow. Mean doses exceeding 25 Gy are associated with severe glandular dysfunction, while exposure below this threshold may permit partial reversibility of symptoms [14]. Notably, irradiation of the parotid ducts themselves appears to have limited relevance for the development of xerostomia [47].

Beyond quantitative loss of saliva, radiation induces qualitative alterations, including shifts in ionic composition and microbial flora. Concentrations of sodium, chloride, magnesium, and calcium rise, while potassium declines. Concurrently, acidogenic and cariogenic bacterial populations increase, contributing to a reduction in salivary pH – frequently approaching 5.0 – and heightened viscosity. These changes may precipitate dysphagia and compromise oral health [48].

In parallel, a reduction in gustatory function is frequently observed. Histological changes, including glandular atrophy, have been reported at doses as low as 10 Gy, often preceding mucositis, with a marked increase in tissue damage at doses above 30 Gy [14].

The persistent lack of salivary secretion substantially impairs quality of life. In addition to loss of taste, patients may report difficulties with speech, mastication, and swallowing. These challenges often lead to dietary adaptations, favoring soft, carbohydrate-rich foods that exacerbate the risk of dental caries [14]. While partial recovery of salivary gland function post-radiotherapy has been described [49], long-term or permanent dysfunction is common, particularly at doses above 25–40 Gy [14].

Xerostomia also has a profound impact on the everyday life of people receiving palliative care, including physical and psychosocial consequences. Speaking is often affected, which can compromise the person’s ability to communicate [50].

Current therapeutic strategies to address xerostomia include the use of salivary substitutes and stimulants. Saliva substitutes aim to replicate the lubricative and protective functions of natural saliva, thereby facilitating speech and deglutition while improving oral comfort. Despite demonstrated benefits on symptom burden and quality of life, their short duration of action necessitates frequent reapplication [51–55]. Pharmacological interventions typically rely on parasympathomimetic agents such as pilocarpine and cevimeline, both of which stimulate residual glandular tissue to enhance salivary flow [56, 57]. Meta-analyses have reported that these agents may be considered as first-line therapy in selected patients [54]. Nonetheless, clinicians must weigh potential side effects and drug interactions carefully [58].

An emerging non-pharmacological modality is transcutaneous electrical nerve stimulation (TENS), which applies low-frequency electrical currents to the periauricular region to stimulate salivary output. While repeated sessions are required for sustained benefit, preliminary studies indicate this approach may alleviate xerostomia symptoms. Optimization of pulse parameters – such as frequency and amplitude – remains an area of active investigation [54, 59–61].

Complementary therapies could also be a useful support in managing symptoms and improving the quality of life. However, solid scientific evidence on their effectiveness and safety has not been found [62]. Still there is some evidence reporting that maintaining regular oral hygiene care may improve xerostomia symptoms [63].

Modern radiotherapy techniques such as Intensity-Modulated Radiotherapy (IMRT) and Volumetric-Modulated Arc Therapy (VMAT) enable conformal dose delivery that spares organs at risk [64]. By modulating dose intensity across multiple beam angles, these approaches significantly reduce irradiation of the parotid glands, resulting in lower incidence and severity of xerostomia [65, 66]. Nevertheless, their effect on oncological outcomes continues to be assessed [65].

Individualized treatment planning allows for sparing of one or both parotid glands when oncologically feasible, with substantial benefits on salivary function preservation. Even partial sparing—limited to one lobe of a gland or stem cell rich regions—has demonstrated a mitigating effect on xerostomia severity and may facilitate post-treatment recovery [67]. These considerations should be integrated into routine radiotherapy planning [68–71].

A potential yet underexplored path involves salivary gland transplantation or ductal rerouting procedures, which have shown promise in preclinical models and select clinical cases. Although technically demanding, these interventions may warrant further investigation in the context of highly conformal radiotherapy planning. Additionally, current classification systems often fail to capture the full impact of xerostomia on daily life; therefore, development of patient-centered xerostomia scoring tools may enhance clinical assessment and guide supportive care [72].

### Mucositis

Oral mucositis, characterized by the inflammation of mucosal linings. According to a meta-analysis by Lee and Galloway [73], the pathogenesis unfolds in a four-phase cascade. Initially, ionizing radiation induces direct cytotoxic effects via single- and double-strand di-ribonucleic acid (DNA) breaks, culminating in apoptosis or loss of proliferative capacity. This effect is particularly pronounced in tumor cells due to their impaired DNA repair mechanisms, thereby aligning with the therapeutic intent of radiotherapy. Additionally, ionizing radiation generates high levels of reactive oxygen species (ROS), overwhelming the cellular antioxidant defenses and further exacerbating DNA injury and cell death. This oxidative stress induces the release of pro-inflammatory mediators, including tumor necrosis factors and interleukins, which not only activate the immune response but also perpetuate tissue damage through secondary ROS release. With progression, epithelial integrity deteriorates, leading to ulcer formation and pseudomembranous lesions. These mucosal breaches become increasingly susceptible to secondary bacterial or fungal colonization – an association frequently observed in clinical practice. Clinically, mucositis manifests as erythema, oedema, and pain, with ulcerations exposing the submucosal layers to the hostile oral environment [73, 74].

Oral mucositis is among the most prevalent adverse effects of radiotherapy in head and neck cancer, occurring during the first weeks of therapy with reported incidences ranging from 80% to 100%. The exact prevalence depends on factors such as total radiation dose and assessment criteria, but nearly all patients experience some degree of mucositis during treatment [74, 75]. Severe forms—classified as grade 3 or 4 according to the WHO mucositis scale—occur in over half of all patients [16, 74]. The grading spans from 0 (no mucositis) to 4 (complete inability to eat due to mucosal breakdown), reflecting the substantial impact on oral intake and overall quality of life. Other ways to assess mucositis (and also other oral affections) are the oral assessment guide (OAG) and Tardieu scale. Although OAG’s simplicity, brevity, and ability to assess individual disorders make it preferable [76].Given this high incidence and clinical burden, mucositis is considered one of the most debilitating side effects associated with head and neck radiotherapy. Swallowing difficulties are common, with nutritional compromise a frequent consequence [75]. An additional contributor to mucosal injury is the presence of metallic dental restorations, such as crowns. These can intensify mucosal burns during irradiation, although the use of intraoral protection devices has proven effective in mitigating this risk [77, 78].

A broad array of therapeutic strategies has been investigated for the prevention and management of oral mucositis. Clinical trials reported these include anti-inflammatory agents, mucoprotectives, herbal and nutritional supplements, and physical interventions such as laser and light therapy [79–84]. Among the pharmacological options, pilocarpine (mucoprotective through saliva production), benzydamine (anti-inflammatory), and zinc chloride preparations (prophylactic by inducing protein synthesis and strengthening membranes) have demonstrated favorable outcomes. Herbal agents, particularly curcumin, have also shown promise in symptom reduction [85–89]. Pilocarpine has additionally been proposed for the prevention of xerostomia, although conclusive evidence remains pending [90]. Adjunctive approaches, as stated in recent meta-analyses, include comprehensive oral care protocols, dietary modifications, topical applications such as honey, this however could aggravate the risk of caries [91]. Moreover, triclosan-containing and other mouth rinses, owing to their antiseptic properties, may support mucosal healing and reduce microbial colonization [92–95].

### Radiotherapy-related tooth loss

Tooth loss in the context of radiotherapy may arise from two distinct but related mechanisms. At the initial dental assessment, teeth with an unfavorable prognosis are often removed based on individualized risk evaluation, aiming to prevent the need for extraction after irradiation and thereby reduce the risk of osteoradionecrosis. In this setting, extractions are undertaken as a preventive measure rather than as a direct consequence of radiation exposure. The most common reasons for these extractions include prophylactic intent (elongated teeth, impacted teeth or retained roots), periodontal impairment and caries [96].

Due to the onset of side effects such as radiation caries, periodontitis or ORNJ, further teeth are lost after radiotherapy [97]. These side effects can influence each other and worsen, for example xerostomia leads to insufficient remineralization [15] leading to an increase in carious lesions, mucositis impedes the ability to maintain oral hygiene [14] increasing attachment loss and periodontitis [43]. Thereby the radiation-induced tooth loss is directly linked to other side effects of radiotherapy and their onset. Since periodontitis, ORNJ and radiation caries are considered late onset side effects, radiotherapy-induced tooth loss can also be counted towards these [97, 98].

The onset of ORNJ is not only a factor to induce radiation related tooth loss but is also linked to the extractions [99]. It is recommended to ensure mucosal healing after extractions before starting radiotherapy to allow the bone to heal with minimal risk of infection.

Tooth loss comes with a decrease in quality of life. Chewing, phonation and aesthetics are functions impacted by tooth loss varying in degree by how many and which teeth specifically are lost [100]. Rehabilitation of lost teeth can be achieved by prosthodontics or implants, although radiotherapy effects on bone and soft tissues increase the risk for rehabilitation failure [101].

### Candidiasis

During radiotherapy patients become more prone to oral infections, most commonly with candida species causing candidiasis. Symptoms can range from none at all to severe pain and inflammation possibly causing discomfort during movement [16]. Overall, the risk of developing a candidiasis is significantly increased in patients receiving radiotherapy with incidences ranging from 12% to 96%. This wide incidence range reflects differences in study design, diagnostic criteria, and patient-related risk factors such as prosthetic use, tobacco consumption, immunosuppression, and degree of hyposalivation [102–104].

Candidiasis can occur all over the oral cavity and the tongue with mainly three appearance forms, pseudomembranous, erythematous and labial occurrence (cheilitis). For diagnostics a swab test with microbiological analysis is most commonly conducted [102].

Treatment of candidiasis varies from local to systemic application of antifungals, as well as managing risk factors. The most commonly used antifungal agents are azoles and polyenes. In mild cases, they are applied topically, whereas in severe cases, systemic administration is indicated, with fluconazole, amphotericin B and nystatin being the most common choice [102, 105]. Intravenous application of antifungals can also be justified in high risk cases or previously failed oral application [102]. A clinical trial reports locally applied miconazole patches have proven to be effective, even showing possibilities in preventative application by keeping the *C. albicans* population in check, this however will not prevent bacterial infections [106].

### Trismus

The musculoarticular complex of the jaw, comprising the temporomandibular joint and four principal muscles (m. temporalis, m. masseter, m. pterygoidius lateralis, m. pterygoidius medialis) is highly vulnerable to radiation-induced impairment. During radiotherapy, this anatomical unit is frequently compromised, resulting in a progressive reduction in mouth opening. Although the precise pathophysiological mechanisms remain incompletely defined, hypotheses range from radiation-induced fibrous dysplasia to disuse-related muscular atrophy [107].

The clinical manifestation of trismus appears to correlate with radiation doses exceeding 40 Gy. Among the muscles most commonly affected are the lateral pterygoid and the masseter [108]. A comprehensive meta-analysis estimated the peak prevalence of trismus to be as high as 44% at six months post-treatment, compared with approximately 17% at the beginning and 32% at 12 months. Beyond this period, prevalence rates appear to stabilize [107]. Recent advancements in radiotherapy, particularly VMAT, have shown potential in reducing the incidence to nearly 10% [109].

Efforts to mitigate trismus have largely focused on dose reduction to the masticatory apparatus. Advanced radiation techniques such as VMAT allow for more precise dose modulation compared to conventional methods, thereby minimizing exposure to vulnerable structures [109–111].

Adjunctive interventions, particularly structured exercise regimens, represent a further approach for prevention and management. These include targeted mobility exercises and, where available, the use of mechanical training devices. Implementation of such exercises during radiotherapy has been associated with a reduction in symptom severity and, in some cases, complete prevention [110, 112–114]. Post-treatment rehabilitation employing similar modalities has likewise demonstrated efficacy in preserving swallowing function and attenuating the progression of trismus [115].

The implementation of structured, proactive physiotherapy programs – initiated before or during radiotherapy – has demonstrated potential in reducing the incidence and severity of trismus [114, 116–119]. However, access to specialized services and reimbursement of medical devices, such as jaw mobilization tools, remain heterogeneous across healthcare systems and may limit broad application [113, 114]. Standardization of referral criteria and integration of physiotherapy programs into oncologic care pathways help with long-term functional preservation.

### Dysphagia through pharyngeal muscle irritation

As well as the musculoarticular complex of the jaw, the muscles of the swallowing complex are also being affected by radiation [120, 121]. This leads to swallowing impairment and overall discomfort of patients receiving radiotherapy. Although no clear pathophysiological process has been shown, it is assumed that the absorbed radiation dose is correlated with the muscular problems of the pharyngeal constrictors and other swallowing muscles [121]. The mean radiation dose for irritation has been shown to be greater than 57 Gy [122].

Generally, the incidence of dysphagia due to pharyngeal muscular problems was shown to be 31%. Risk factors for higher irritation rates are old age and female biological gender [123].

The treatment of dysphagia mostly relies on swallowing exercises, manual massaging therapy or acupuncture, although evidence for these strategies has not been found [124]. The University of Iowa has also introduced a medical device measuring the strength of tongue pressure to the palate with the aim of strengthening the tongue and helping with swallowing. Medical devices such as this provide biofeedback, which helps the patient visualize their progress and helps with motivation [125]. All of these strategies require good patient compliance to be effective [124, 125].

As for prevention, the main goal is to reduce the mean dosage through dysphagia-optimized intensity-modulated radiotherapy (DO-IMRT), which has been shown to improve swallowing function by lowering mean dosage to the pharyngeal constrictors and other swallowing muscles to below 50 Gy [126].

### Osteoradionecrosis

Osteoradionecrosis of the jaw (ORNJ) represents a rare but severe and potentially debilitating complication observed in patients undergoing radiotherapy for head and neck cancer [127]. The most common location of ORNJ is the lower jaw. This is thought to result from its more frequent and extensive inclusion in radiation target volumes, as well as its limited blood supply and smaller surface area available for denture support [128].

According to the new consensus statement of an interdisciplinary Delphi study bone does not need to be exposed in order to be classified as ORNJ, rather bone death in an irradiated field caused by a lack of blood flow. Diagnostic parameters for this definition include a probing-to-bone test and mucosal status, radiographic imaging and vascular imaging [129]. Symptoms include pain, swelling, trismus, halitosis, neuropathic pain, local anesthesia, dysgeusia, impaired food clearance, secondary infections and a substantial decrease in quality of life [130, 131]. Severe cases may progress to fistula formation or pathologic fractures [132]. The increasing number of patients receiving antiresorptive or antiangiogenic agents in combination with radiotherapy is of particular concern, which further elevate the risk of osteonecrosis independent of radiation alone [133]. These agents further increase the risk of jaw necrosis, which is referred to as medication-related osteonecrosis of the jaw (MRONJ), complicating both prevention and treatment. A detailed medication history and interdisciplinary treatment planning are critical to stratify risk and avoid invasive dental procedures during periods of heightened vulnerability [134]. Concurring chemotherapy, immunotherapy or targeted agents can make side effects of radiotherapy worse. Chemotherapy has impact on cell replication and tissue regeneration, impeding mucosal healing and causing inflammation [135], Immunotherapy on salivary flow and targeted agents can influence salivary flow and cause mucosal inflammation [136]. A careful anamnesis is necessary to check for combined therapy and to be considerate of combined effects.

Reported incidence rates vary substantially across studies, ranging from 3.2% [99] to 8.8%. This reflects differences in radiation dose, tumor location, dental extraction timing, and concurrent medication use (e.g., antiresorptives or antiangiogenics) [137]. Notably, patients undergoing dental extractions or other invasive procedures shortly before to radiotherapy exhibit a consistently higher risk [99, 137–139].

Despite prophylactic intent, extractions – frequently performed before radiotherapy – can delay initiation of radiotherapy due to the requirement for complete mucosal healing. Comparative studies assessing the timing of extractions (pre- versus post-radiotherapy) found no statistically significant difference in ORNJ occurrence, however, pre-radiotherapy extractions may introduce delays [17, 99, 137]. Among affected individuals, the median time of onset of ORNJ was approximately 5 months [137].

Risk stratification further implicates radiation dose as a critical determinant. While one investigation identified a marked increase in ORNJ incidence above 50 Gy mean dose [140], others report that parameters such as mean dose (Dmean) and biologically effective dose (BED2) yield a more nuanced understanding, complicating the identification of a strict threshold [141]. Nevertheless, a consensus persists that mandibular dose should be minimized wherever feasible.

Given the importance of dose-related bone changes, radiological imaging plays a pivotal role in the diagnosis, staging, and monitoring of osteoradionecrosis. Panoramic radiographs, computer tomography (CT) and cone-beam CT (CBCT) are considered first-line modalities due to their high sensitivity for cortical disruption, bone fragmentation, trabecular rarefaction, and sequestrum formation. Characteristic CT findings include mixed lytic-sclerotic lesions, loss of cortical continuity, and dense bony sclerosis [130, 142, 143]. Advanced ORNJ may also present with pathological fractures, bicortical involvement, and gas-like lucencies suggestive of superimposed osteomyelitis [144]. Bony sclerosis reflects a pathological response of devitalized bone to chronic radiation-induced hypoxia, osteocyte loss, and impaired remodeling, and is considered a hallmark imaging feature of ORNJ. It is typically absent in recurrent tumors. Conversely, the presence of solid or cystic enlarging masses should raise suspicion for recurrence [145].

Magnetic resonance imaging (MRI) plays a significant role in the early detection of ORNJ, even in the absence of mucosal breakdown. Low signal intensity on T1-weighted images combined with heterogeneous or high T2 signal, with or without contrast enhancement, typically reflects bone marrow edema and early fibrosis [144]. MRI is also superior for assessing the extent of soft tissue inflammation, involvement of the masticatory musculature, and perilesional edema, findings that correlate with clinical symptoms such as trismus or pain [146]. As stated in the consensus definition, devascularization plays a critical role in the onset of ORNJ. These vascular deficiencies can be detected with MRI and thus be taken into consideration when diagnosing ORNJ [129].

Positron Emission Tomography/Computed Tomography (PET/CT) provides valuable metabolic information that complements morphologic imaging. ORNJ typically shows moderate, non-focal gluorodeoxyglucose (¹⁸F-FDG) uptake due to chronic inflammation, whereas tumor recurrence presents with sharply localized, intense uptake. However, inflammatory and infectious changes such as osteomyelitis can also cause increased tracer accumulation, potentially limiting specificity. In ambiguous cases, minimally invasive biopsy may be required for definitive diagnosis [145, 146].

Multiple preventive strategies have been explored to reduce the incidence of ORNJ. Despite the biological plausibility of platelet-rich fibrin to enhance alveolar healing after extractions, no significant benefit has been observed in randomized comparisons for preventing ORNJ [147–149]. Similarly, adjunctive treatments such as hyperbaric oxygen (HBO), pentoxifylline–tocopherol combinations, and prophylactic antibiotics have failed to demonstrate consistent preventive efficacy. However, antibiotic use remains favored for its cost-effectiveness [150, 151]. Photobiomodulation therapy has shown promise in accelerating mucosal healing, potentially reducing delays in the initiation of radiotherapy [152, 153].

During treatment planning, mechanical protection strategies are frequently employed to shield critical structures or increase the distance between high-risk tissues and radiation-sensitive dental restorations. These include radiation carriers, splints, and customized protective devices – each contributing to mandibular dose reduction when correctly implemented [78].

Established ORNJ remains therapeutically challenging, with management strategies ranging from conservative to surgical. Sequestrectomy remains the mainstay intervention for necrotic tissue removal, although extensive resections often result in structural compromise and tooth loss [134]. In severe cases, these surgical interventions can range from partial resection to complete removal and reconstruction of the entire jaw [154, 155].

HBO therapy, designed to enhance oxygenation and thereby support tissue regeneration, has been extensively studied; however, robust evidence remains elusive due to limited cohort sizes [156, 157]. A meta-analysis reports that select patients may benefit from HBO, though the therapy is resource-intensive and carries risks including barotrauma and visual disturbances [157].

Pharmacological management with pentoxifylline–tocopherol (PENTO) has gained increasing interest. Administered at a daily dose of 800 mg pentoxifylline and 1000 IU vitamin E daily over a six-month period, this regimen has shown therapeutic potential, although confirmatory studies remain warranted [158].

Emerging data present the immunological microenvironment as a potential contributor to both the pathogenesis and resolution of ORNJ. Immune cell dynamics, in concert with osteoblast-mediated regeneration, may provide novel diagnostic markers and therapeutic targets. Innovations such as biomaterial-based scaffolds, bioprinting technologies, monoclonal antibodies, and gene therapies are currently under exploration, holding promise for future individualized approaches to bone repair [159].

## Discussion

**Fig. 4:**
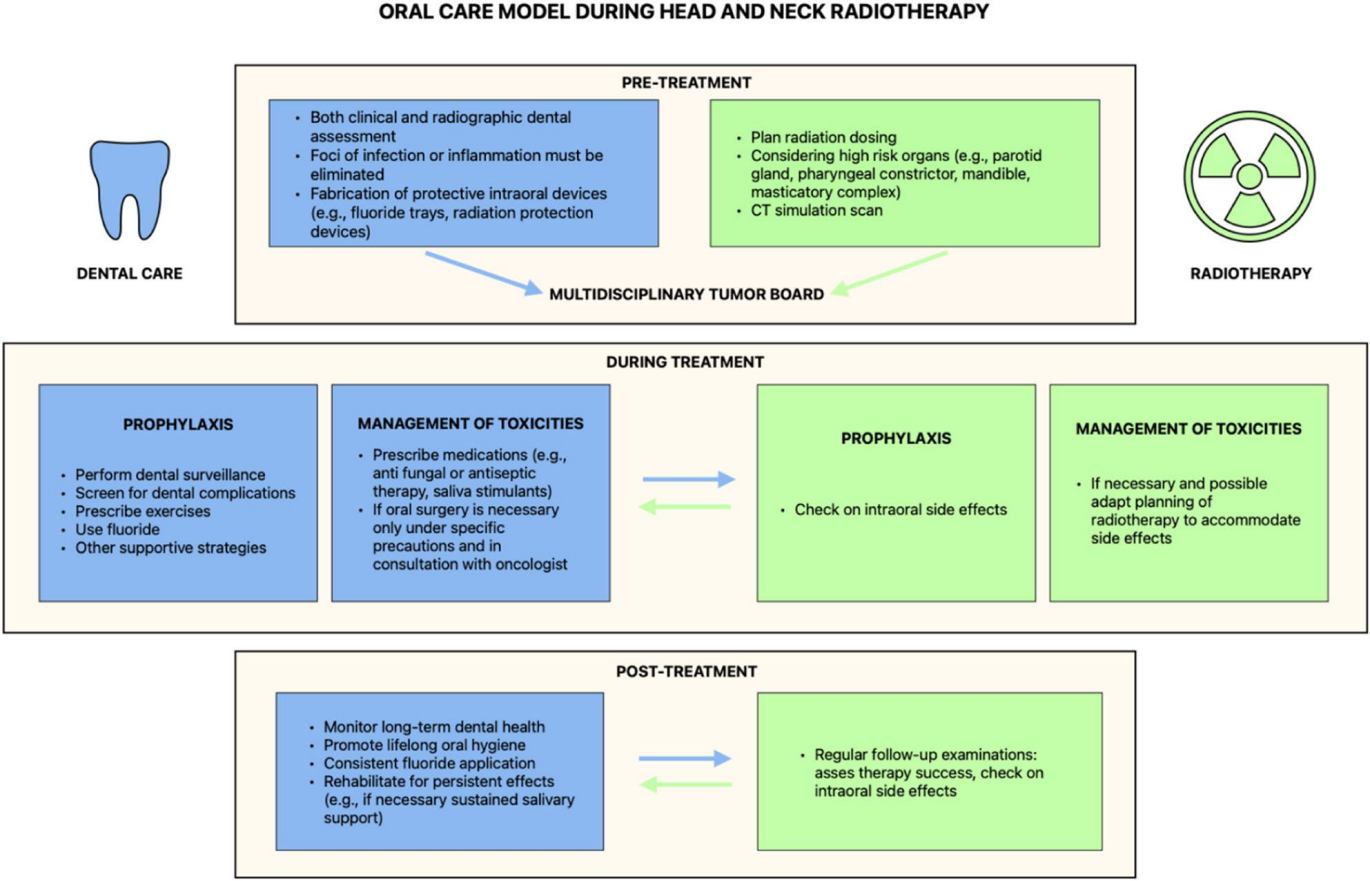
Summary of the integrative care model discussed above

Radiotherapy for head and neck cancer carries a substantial risk for oral complications. Although mucositis, xerostomia, candidiasis, radiation-induced dental caries, periodontitis, trismus, and osteoradionecrosis are well-characterized adverse effects, their prevention and management remain insufficiently embedded in routine oncologic workflows. Drawing upon current evidence and interdisciplinary consultation, we outline a comprehensive care model that incorporates dental expertise at all stages of radiotherapy, shown in Fig. 4.

Even in palliative care situations there are models of integrating a dentist into the interdisciplinary palliative care team. With very little dental effort and simple ward and bedside treatments, significant improvements in the oral symptom burden of critically ill palliative patients can be achieved. This contributes to improved care status, relief of distressing symptoms, and ultimately improved quality of life in this often highly affected patient population [160].

Preventive strategies remain a fundamental component of care. Lifelong oral hygiene, consistent fluoride application, and sustained salivary support are essential [18, 45]. When oncologically feasible, treatment plans should prioritize sparing of salivary glands and mandibular structures [68, 70].

An interdisciplinary care model that integrates dental professionals throughout the radiotherapy trajectory is not only feasible but essential to optimize outcomes, preserve oral function, and maintain quality of life. Implementation on a broader scale requires institutional commitment and structural support.

Current barriers include fragmented communication between oncology and dental care, particularly in outpatient settings. Establishing standardized referral pathways, interoperable documentation systems, and multidisciplinary tumor boards will be pivotal.

Robust prospective data on integrated dental-oncologic care remain scarce. While present recommendations are grounded in pathophysiologic rationale and retrospective evidence, prospective evaluation is essential to delineate optimal timing, intensity, and components of dental interventions. The DentalRad registry at University Hospital Düsseldorf represents a vital next step toward generating real-world evidence to inform clinical guidelines and advance interdisciplinary models of care.

### Strategies to prevent radiation-induced oral toxicities: interdisciplinary treatment plan

Comprehensive dental evaluation and intervention represent essential components of preparatory care in patients undergoing head and neck radiotherapy. Pre-treatment dental clearance aims to eliminate potential sources of infection, prevent irreversible damage to the dentition and supporting structures, and mitigate radiation-induced toxicities through structural, chemical, and behavioral interventions.

All patients should undergo a thorough dental examination prior to radiotherapy planning, including clinical inspection, panoramic and intraoral radiography, and, where clinically appropriate, cone-beam CT. Coordination between dental and radiation oncology teams is crucial at this stage to align treatment goals and assess anatomical proximity between oral structures and radiation fields.

Teeth with poor prognosis – including those affected by extensive caries, (peri-) apical pathology, or advanced periodontal disease – should be extracted 14–21 days prior to the initiation of radiotherapy if clinically feasible to ensure complete mucosal healing. Extraction indications include non-restorable teeth, severe periodontal disease including probing depth of >6 mm, furcation involvement >3 mm, mobility of II-III degree and particularly the combination of these conditions as well as retained root fragments. Special consideration should be given to teeth within anticipated high-dose areas (> 50 Gy), where even marginally compromised teeth may contribute to later complications. Primary closure is recommended, particularly in high-dose regions, and post-operative imaging should be obtained prior to CT simulation. The use of systemic antibiotics remains debated but may be considered in selected high-risk cases.

In contrast, teeth that are stable but affected by moderate caries or gingivitis should be restored and stabilized, using materials such as fluoride-releasing glass ionomer cements or composite resins. Periodontal debridement and oral hygiene instruction should be provided to all patients, regardless of restorative status.

To counteract the elevated caries risk associated with xerostomia and direct radiation injury, patients should begin daily fluoride therapy using custom-made upper and lower trays filled with 5,000 ppm fluoride gel, starting approximately one week before treatment. Trays should be worn for 5–10 min daily, and the regimen continued throughout treatment. Supplementary measures include high-fluoride toothpaste, alcohol-free rinses, and salivary substitutes. A close check-up and prophylaxis routine at the dentist should also be established during and after radiotherapy.

Beyond chemical protection, intraoral positioning devices serve both dosimetric and protective functions. In cases of unilateral irradiation, mandibular displacement devices or open-bite blocks may be employed to increase the spatial distance between the target volume and non-involved oral structures if clinically applicable and not hindering radiotherapy. These must be fabricated prior to simulation and worn during both planning and daily radiotherapy treatment to ensure reproducibility.

Special consideration is warranted for patients with metallic dental restorations such as amalgam, gold, or metal-ceramic crowns, which may generate scatter radiation leading to localized mucosal overdosing. In such cases, the fabrication of customized shielding splints made of tissue-equivalent materials (e.g., thermoplastics or silicone) can physically separate mucosal surfaces from metal interfaces. This simple intervention has been shown to reduce the risk of ulceration and enhance mucosal tolerance during treatment.

Post-treatment follow-up is essential to preserve oral health, detect complications early, and maintain quality of life. Structured dental surveillance should occur within the first three months, followed by biannual or annual assessments depending on individual risk. Patients should be re-evaluated for trismus, periodontal deterioration, mucosal lesions, and signs of osteoradionecrosis. Functional rehabilitation—including jaw mobility exercises and swallowing therapy—should be initiated as early as feasible and continued throughout survivorship.

## Conclusion

A short summary for each side effect discussed above is provided:

Radiation-induced caries, typically affecting smooth tooth surfaces, is strongly associated with xerostomia and exceeds 25% in prevalence, with some cohorts exceeding 50%. Risk factors such as oral hygiene protocols and degree of xerostomia influence this prevalence. Preventive regimens include fluoride applications, salivary support, and professional dental care. Restorative options such as composite resins and fluoride-releasing glass ionomer cements are frequently employed [14, 16, 26, 29, 38].

Periodontitis, affecting up to 70% of patients, requires management across the entire treatment timeline. Diagnosis can be reached through clinical parameters such as clinical attachment loss or through biomarkers such as CARWL [44]. Standard periodontal therapy—including scaling and root planing—remains effective. Preventive strategies involve rigorous oral hygiene, addressing modifiable risk factors, and minimizing radiation dose to periodontal tissues [35, 42, 45, 46].

Xerostomia results from radiation exposure to salivary glands, particularly the parotids. Evidence suggests a mean parotid dose threshold of around 25 Gy, below which significant functional recovery can occur; exceeding this threshold, however, results in markedly reduced salivary flow with little or no long-term restitution. Management includes pilocarpine, saliva substitutes, and TENS, whereas IMRT and VMAT offer substantial sparing potential when strategically planned [14, 48, 54, 65, 70].

Oral mucositis, affecting 80–100% of patients, stems from radiation-induced DNA damage, oxidative stress, and inflammatory responses. Management involves pharmacologic agents (e.g., pilocarpine, zinc chloride, benzydamine), herbal remedies, and meticulous oral care. Due to the mucosa being in the field of radiation in most cases, preventive strategies remain limited, though antiseptic rinses like triclosan may reduce secondary infections [73, 74, 90, 94].

Radiotherapy induced tooth loss is closely linked to caries, ORNJ and periodontitis. Teeth can be lost before radiotherapy through planned extractions of teeth deemed no longer sufficiently treatable or potential cause of infection in the future [96]. Decision factors for extraction are caries status, periodontal status, impaction or remaining roots or involvement in inflammatory processes. Tooth loss after radiotherapy is mostly caused by or induced by other side effects such as radiation caries, radiation induced periodontitis or ORNJ [97, 98], ORNJ also being influenced by the loss or extraction of teeth in return [99]. Treatment options are similar to treatment of regular tooth loss, such as prosthodontics or surgical implants, but are limited by the effects of radiotherapy on the bone and mucosal tissues, making rehabilitation more difficult [101].

Candidiasis, which affects up to 96% of patients, is associated with hyposalivation and immunosuppression. Management includes local or systemic antifungals such as miconazole. Preventive measures range from antiseptic rinses to antifungal patches and personalized oral hygiene strategies [102, 103, 106].

Trismus, with a prevalence stabilizing around 32% at one-year post-treatment, is best managed through structured exercise protocols supported by medical devices. Prophylactic interventions include early jaw mobility exercises and dose sparing to the temporomandibular joint and associated musculature [107, 109, 113–115].

Dysphagia, linked to radiation-induced injury of pharyngeal musculature—especially at doses > 57 Gy—affects roughly one-third of patients. Exercise-based interventions, acupuncture, and biofeedback have demonstrated efficacy. Prophylactic strategies such as dysphagia-optimized IMRT (DO-IMRT), with mean doses < 50 Gy to swallowing structures, may significantly reduce functional decline [122–124, 126].

ORNJ remains one of the most severe long-term toxicities, with onset typically occurring five months post-treatment. Risk factors include high-dose radiation (> 50 Gy) and previous dental surgery. Established therapies comprise conservative management, sequestrectomy, and hyperbaric oxygen therapy. Investigational approaches such as photobiomodulation and prophylactic antibiotics hold promise, as do biomarker-based diagnostics and biomaterial scaffolds for reconstruction of lost bone material [134, 137, 140, 147, 152, 157, 159].

Radiological imaging plays a supportive but critical role in the diagnosis, staging, and follow-up of osteoradionecrosis. Advanced modalities such as cone-beam CT, CT, MRI and PET/CT enable differentiation between necrotic bone, tumor recurrence, and inflammatory changes. CT and CBCT are highly sensitive for detecting cortical disruption, fragmentation, and sequestra, while MRI excels at visualizing bone marrow edema and soft tissue involvement [143, 144]. As a future perspective, the ClinRad classification system proposed by Watson et al. [161]. represents a promising step toward standardized radiologic staging. By incorporating imaging morphology and clinical risk factors, ClinRad improves prediction of complications such as pathologic fracture or need for resection. Though not yet widely implemented, its integration into recent American Society of Clinical Oncology (ASCO) guidelines highlights its rising relevance in clinical and research settings [142, 161].

A thorough pre-treatment dental assessment is imperative. Foci of infection or inflammation should be eliminated, and carious lesions managed appropriately [18]. Early communication between dental and radiation oncology teams facilitates targeted dose optimization, especially in high-risk intraoral zones. The fabrication of protective intraoral devices—including positioning aids and fluoride trays—should precede therapy initiation [25, 33, 78, 162, 163]. Simultaneously, patients require anticipatory counseling regarding oral hygiene and the management of potential toxicities [26, 90].

Throughout radiotherapy, patients benefit from routine dental surveillance ensuring early detection of emerging complications [18, 45]. This monitoring should encompass mucosal inspection, salivary gland assessment, caries surveillance, and periodontal evaluation. Preventive exercises targeting the masticatory apparatus are recommended to mitigate trismus. Given their frequent patient contact, radiation oncologists should routinely screen for oral symptoms and coordinate timely referrals.

Post-treatment, structured rehabilitation programs are critical. Reversible toxicities—such as trismus and xerostomia—respond to physical and pharmacologic interventions [54]. Dental caries, periodontitis, and ORNJ necessitate long-term dental monitoring [18, 45]. Advances such as microbiome profiling and biomarker analytics may enhance early detection and individualized management [159]. We recommend to closely monitor patients through their radiotherapy and follow a dental care program to ensure optimal outcome and quality of life.

## Limitations

This scoping review has limitations which should be considered when interpreting the findings.

Only papers published in English language were taken into the review process. As a result, potentially relevant articles in other languages were not considered. This proves a risk of language bias and limits the global representativeness of this paper particularly in fields of research in non-English speaking countries.

Papers published before 1999 were also excluded from the review process. While this decision was made to ensure contemporary relevance and shift focus to more recent diagnostic and treatment standards it might exclude studies which contributed to the foundation of topics discussed in this paper which might not be fully captured.

The search was conducted on two databases, PubMed (MEDLINE) and the Cochrane Library, which may have led to incomplete retrieval of relevant studies. This may introduce publication bias and limit the breadth of evidence.

As with all scoping reviews, the objective was to map the available literature rather than critically appraise methodological quality or perform quantitative synthesis. This review did not conduct a formal strength-of-evidence or risk of bias analysis, although studies with transparent methodology, appropriate cohort sizes and clear clinical relevance were prioritized during the review process. Despite this we aim to provide a structured overview of the current research within our limitations and identify key gaps to inform future research.

## Data Availability

Our data was sourced via the online databases [PubMed] (https://pubmed.ncbi.nlm.nih.gov) and [Cochrane Library] (https://www.cochranelibrary.com). All data supporting the findings of this study are derived from previously published literature, no new datasets were generated. The articles from which the data was extracted are referenced below, the time of analysis was between September 2024 and November 2025. Further data requests are available through the corresponding author on reasonable request.

## Abbreviations

TENS: Trans cutaneous nerve stimulation
IMRT: Intensity-Modulated Radiotherapy
VMAT: Volumetric-Modulated Arc Therapy
DNA: Di-ribonucleic acid
ROS: Reactive oxygen species
OAG: Oral assessment guide
DO-IMRT: Dysphagia-optimized intensity-modulated radiotherapy
ORNJ: Osteoradionecrosis of the jaw
MRONJ: Medication related osteonecrosis of the jaw
Dmean: Mean dose
BED2: biologically effective dose
CT: Computer tomography
CBCT: Cone-beam CT
MRI: Magnetic resonance imaging
PET/CT: Positron Emission Tomography/Computed Tomography
¹⁸F-FDG: Non-focal gluorodeoxyglucose (18 F)
HBO: Hyperbaric oxygen
PENTO: Pentoxifylline-tocopherol
ASCO: American Society of Clinical Oncology
